# Locally advanced non-small cell lung cancer with negative or low programmed death ligand 1 expression: a prognostic factor analysis of real-world data after the PACIFIC trial

**DOI:** 10.1186/s13014-025-02733-5

**Published:** 2025-10-16

**Authors:** Tairo Kashihara, Yuko Nakayama, Kae Okuma, Ayaka Nagao, Kana Takahashi, Tomoya Kaneda, Yuko Kubo, Kimiteru Ito, Satoshi Nakamura, Hiroyuki Okamoto, Yasushi Yatabe, Masahiko Kusumoto, Yuichiro Ohe, Hiroshi Igaki

**Affiliations:** 1 Department of Radiation Oncology, National Cancer Center Hospital, 5-1-1 Tsukiji, Chuo-ku, Tokyo, 104-0045 Japan; 2 Department of Thoracic Oncology, National Cancer Center Hospital, 5-1-1 Tsukiji, Chuo-ku, Tokyo, 104-0045 Japan; 3 Department of Diagnostic Radiology, National Cancer Center Hospital, 5-1-1 Tsukiji, Chuo-ku, Tokyo, 104-0045 Japan; 4 Department of Diagnostic Pathology, National Cancer Center Hospital, 5-1-1 Tsukiji, Chuo-ku, Tokyo, 104-0045 Japan

**Keywords:** Radiotherapy, Immunotherapy, Interstitial lung disease, Prognosis, Mean heart dose

## Abstract

**Background:**

Real-world data on patients with locally advanced non-small cell lung cancer (LA-NSCLC) treated with chemoradiotherapy have been reported; however, prognostic factors in patients with negative or low programmed cell death ligand 1 (PD-L1) expression are unclear. Therefore, we aimed to explore prognostic factors in patients with LA-NSCLC with negative or low PD-L1 expression using real-world data obtained after the PACIFIC trial.

**Methods:**

Patients with LA-NSCLC with negative or low PD-L1 expression who received definitive radiotherapy at our institution between March 2017 and May 2022 were included. Competing risk analyses were used to evaluate cumulative incidence of cancer-specific death (CI-CSD), cumulative incidence of recurrence (CI-R), cumulative incidence of distant metastasis (CI-DM), and cumulative incidence of in-field recurrence (CI-IFR).

**Results:**

The study included the data of 130 patients. Median follow-up was 37 (range, 2–72) months after radiotherapy initiation; 54 (41.5%) patients died, among whom 49 died of lung cancer, and 75 (57.7%) received durvalumab. Multivariate analyses revealed that interstitial lung abnormality (ILA) score ≥ 1 was a significant factor of shorter OS and higher CI-IFR, and a mean heart dose ≥ 7.5 Gy showed a trend toward significance for shorter OS., Internal high-dose volumetric modulated arc therapy was associated with lower CI-R, whereas poor baseline performance status correlated with higher CI-DM. Notably, durvalumab administration was marginally associated with lower CI-R.

**Conclusions:**

Pre-treatment ILA and high mean heart doses negatively impacted OS in patients with LA-NSCLC with negative or low PD-L1 expression according to real-world data obtained after the PACIFIC trial.

**Supplementary Information:**

The online version contains supplementary material available at 10.1186/s13014-025-02733-5.

## Background

Concurrent chemoradiotherapy (CCRT) has long been the standard treatment for locally advanced non-small cell lung cancer (LA-NSCLC). The PACIFIC trial demonstrated that consolidation therapy with durvalumab following CCRT significantly improves progression-free survival (PFS) and overall survival (OS) [1–4]. After the PACIFIC trial, real-world data of adjuvant durvalumab following CCRT in patients with LA-NSCLC have been reported multiple times [5–9]. PD-L1 expression, measured by tumour proportion score (TPS), has been suggested as a potential biomarker for the efficacy in patients with LA-NSCLC treated with CCRT plus durvalumab [10], and no benefit on OS was observed in those with negative PD-L1 expression [11]. However, prognostic factors in patients with negative or low PD-L1 expression are yet to be determined. Moreover, the RTOG0617 trial found that dose volume histograms (DVHs) for the heart were associated with OS [12]. Nevertheless, the impact of DVH information, including the heart, on treatment outcomes in patients with LA-NSCLC after the PACIFIC trial remains unclear. Therefore, this study aimed to evaluate real-world prognostic factors—including cardiac and pulmonary dose-volume parameters—in patients with LA-NSCLC and negative or low PD-L1 expression, to better inform clinical decision-making in this distinct population.

## Methods

### Data collection

The data of patients with LA-NSCLC who received definitive radiotherapy at our institution from March 2017 to May 2022 were retrieved from our radiotherapy database. PD-L1 expression status was investigated, and the patients with PD-L1 TPS ≥ 50% or unknown were excluded from the analyses. At least one radiologist and oncologist staged the patients according to the eighth edition of the TNM classification. The factors examined at the initiation of radiotherapy were as follows: Zubrod performance status (PS) at the initiation of radiotherapy, sex, age, smoking history, clinical stage, pathological diagnosis, PD-L1 TPS, clinically actionable genetic mutations (e.g., EGFR, BRAF, KRAS, ALK, and RET), excluding variants without approved targeted therapies, interstitial lung abnormality (ILA) scores, radiotherapy technique (intensity-modulated radiotherapy [IMRT]), the use of intentional internal high-dose volumetric modulated arc therapy (IIHD VMAT), administration of durvalumab, and DVH of the lungs and heart. The ILAs were evaluated by at least one radiologist and one radiation oncologist using the most recent pre-radiotherapy diagnostic computed tomography (CT) images. Diagnostic CT images were acquired at 1–3-mm thickness in the inspiratory phase. The scoring method of the ILA score was previously reported and is as follows: 0, no ILAs; 1, ILA changes without honeycomb (microcysts, fine reticular opacities, and ground-glass attenuation); and 2, honeycombs.^13^ Furthermore, PD-L1 immunostaining was automatically performed with a monoclonal antibody (22C3; Dako, CA, USA). PD-L1 TPS was calculated as the proportion of tumour cells positive for PD-L1 staining in formalin-fixed paraffin-embedded specimens. The Oncomine Dx Target Test was used for molecular testing in our cohort. However, the decision to perform molecular testing prior to treatment was made at the discretion of the treating physician, based on the clinical context, histology, and availability of adequate tissue samples.

### Treatment methods

**Fig. 1:**
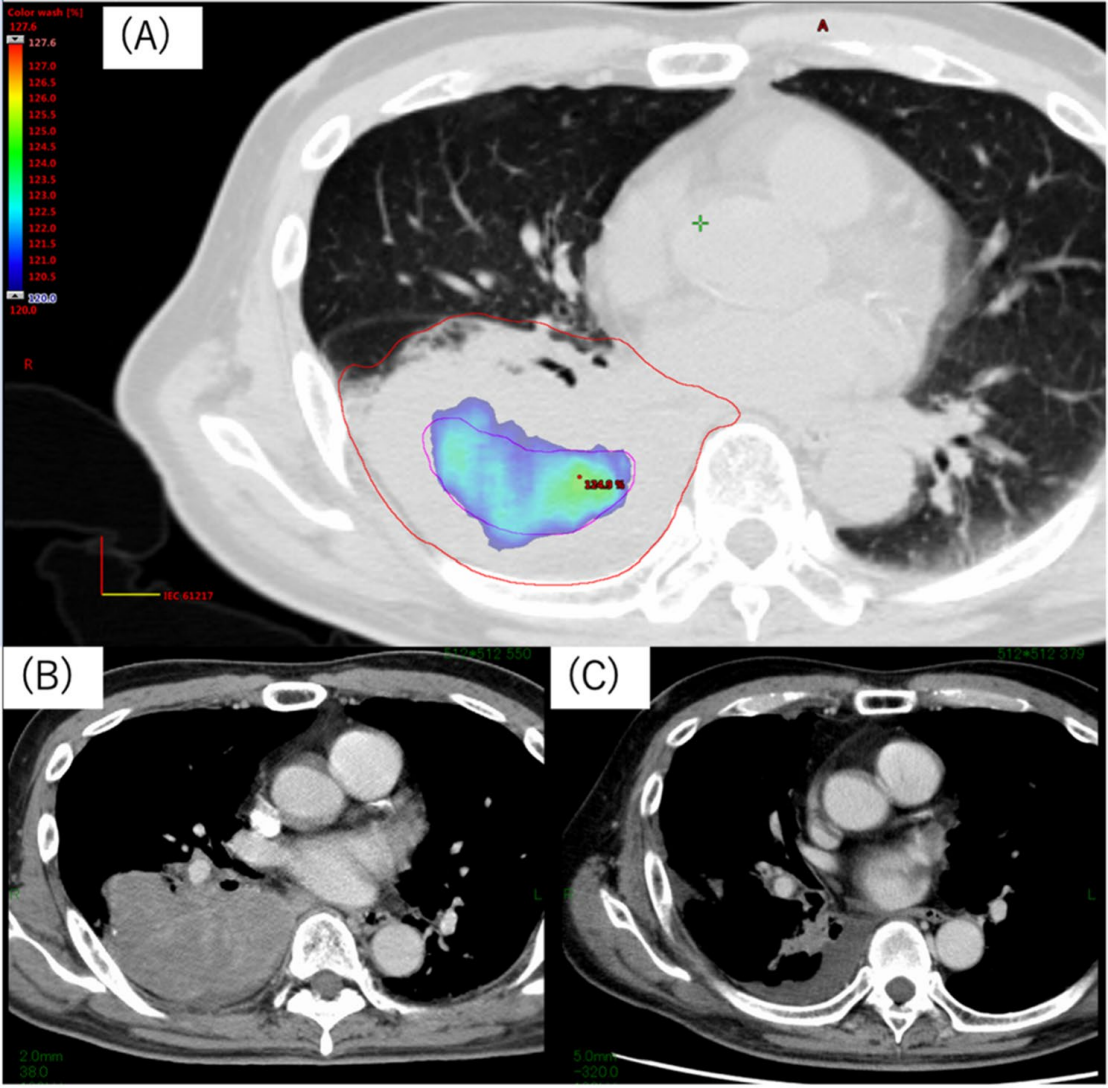
A patient receiving IIHDP VMAT. (**a**) An example of IIHDP VMAT. Dose-colour-wash area represents 120% of the prescription dose. (**b**) A CT image before the initiation of chemoradiotherapy, (**c**) A CT image acquired 5 months after the completion of chemoradiotherapy. CT, computed tomography; IIHDP VMAT, internal high-dose volumetric modulated arc therapy

All eligible patients were considered for chemoradiotherapy prior to treatment, based on a multidisciplinary decision by a radiation oncologist, a respiratory surgeon, and a respiratory physician. Radiotherapy methods have been previously described [13]. Treatment planning CT images were obtained with a slice thickness of 2 mm and 3 mm for IMRT and three-dimensional conformal radiotherapy (3DCRT), respectively. Margins from gross tumour volumes to clinical target volumes (CTVs) for the primary tumours and the lymph nodes were 0–5 mm. Margins from CTVs to planning target volumes were 5–10 mm. Notably, prophylactic CTV was designed at the discretion of the attending radiation oncologists [14]. Additionally, IIHD VMAT was permitted for the large primary and/or lymph node lesions (see Fig. 1) [15, 16]. All patients received radiotherapy with 6 or 10 MV X-rays in linear accelerators (Varian, California, USA). Radiotherapy was administered at 2 Gy/day, five days/week. The prophylactic CTVs were irradiated with 3DCRT up to 40 Gy or with IMRT up to 46–51 Gy using simultaneous integrated boosting (SIB). Durvalumab administration was determined by the physicians based on the results of blood tests, CT images, and medical examinations.

### Assessment of outcomes

Survival data, including OS, cumulative incidence of cancer-specific death (CI-CSD), cumulative incidence of recurrence (CI-R), cumulative incidence of distant metastasis (CI-DM), and cumulative incidence of in-field recurrence (CI-IFR) were collected in May 2023. Competing risk analyses were used to calculate CI-CSD; deaths from causes unrelated to lung cancer were considered competing risks. They were also used to calculate CI-R, CI-DM, CI-IFR; all deaths were considered as competing risks. The completion date of definitive radiotherapy was used as the initial date in the survival analysis. Moreover, subgroup analyses of the prognostic factors were performed in patients with negative PD-L1 expression.

### Statistics

The Cox proportional hazards regression models were used to examine the prognostic factors of the survival analyses. Continuous variables were dichotomized using their median values for univariate analysis. Smoking history was categorized as a binary variable (ever-smoker vs. never-smoker) based on patient-reported history at the time of treatment initiation. In multivariate analysis, factors that were a p-value of < 0.1 in univariate analysis were included. Because IMRT and IIHD VMAT are related factors, only the factor with the stronger correlation regarding hazard ratio (HR) was included in multivariate analysis. If there was only one factor with a p-value of < 0.1 in univariate analysis, the factor with the strongest correlation based on HR was included in a multivariate analysis. Statistical significance for multivariate analysis was set at p-values of < 0.05. OS was analysed using IBM SPSS Statistics for Mac, V 26.0, whereas the other survival data were analysed with EZR [17], a modified version of the R commander. Statistical analysis was conducted in July 2023. The risk factors for radiation pneumonitis grade ≥ 2 were similarly evaluated using competing risk analysis. This additional analysis was performed in August 2025 using Stata BE 18.0.

## Results

**Fig. 2:**
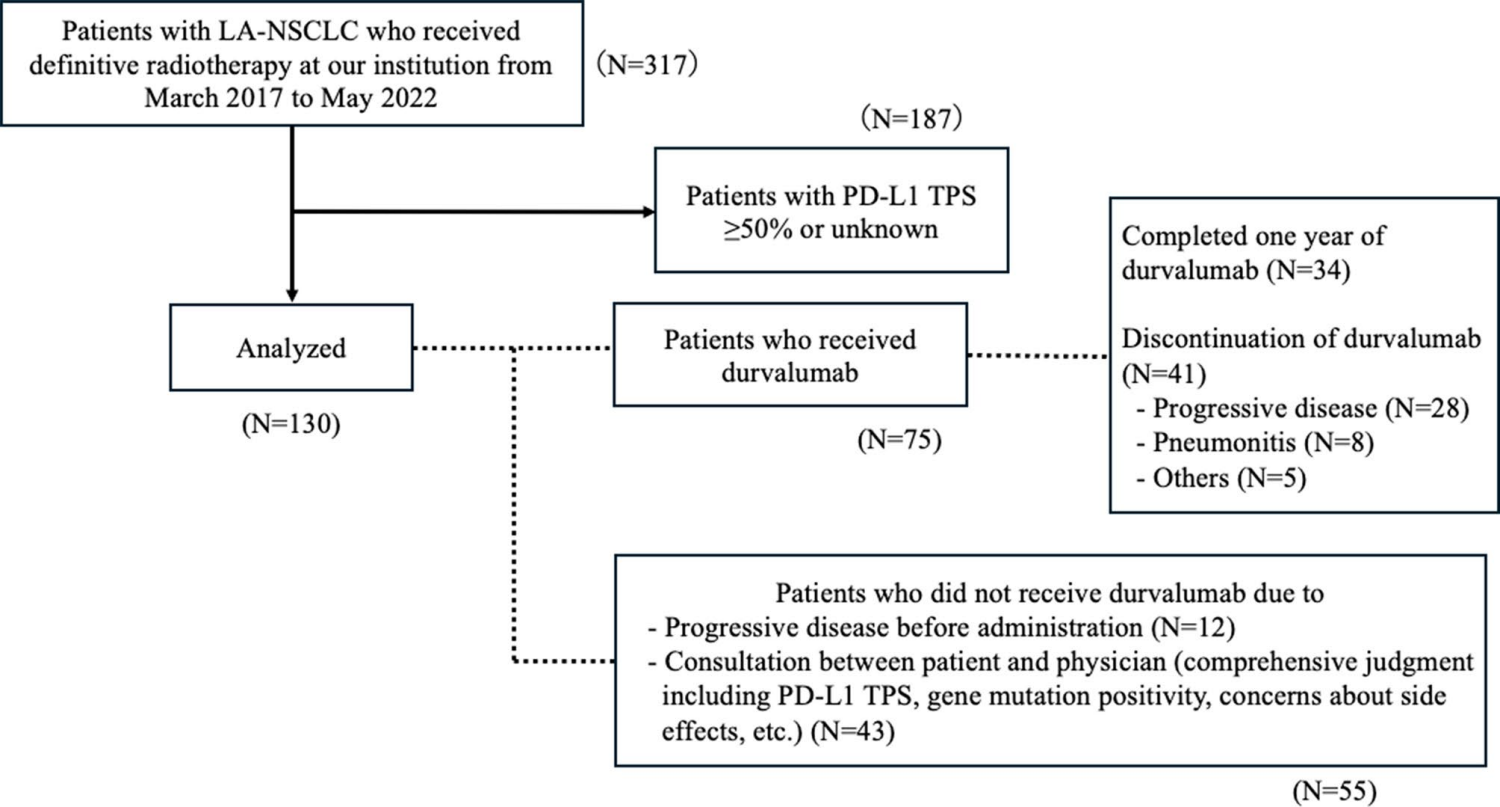
Patient flowchart and details of durvalumab administration. Flowchart of patient inclusion and durvalumab administration. Among 130 patients with PD-L1 TPS < 50%, reasons for durvalumab completion, discontinuation, or non-administration are shown. PD-L1, programmed cell death ligand 1; TPS, tumour proportion score

Among the 317 patients who received definitive radiotherapy, those with PD-L1 TPS ≥ 50% or unknown were excluded, and 130 were included in this study. The patient selection process and reasons for durvalumab completion or discontinuation are illustrated in Fig. 2. Table 1 represents the patient and treatment characteristics. The median age of the patients was 67 (range: 25–84) years. Fifty-four (41.5%) patients had negative PD-L1 expression. Nineteen (14.6%) patients had an ILA score ≥ 1. Additionally, clinically actionable genetic mutations were detected, undetected, and unknown in 26 (20.0%), 84 (64.6%), and 20 (15.4%) patients, respectively. Clinically actionable genetic mutations included 16 *EGFR* mutations, six *ALK* fusions, three *KRAS* mutations, and one *RET* fusion. Additionally, 124 (95.4%) patients were irradiated with 60–66 Gy in 2 Gy/day. Seventy-five (57.7%) patients received a median of 14.5 cycles of durvalumab. No patients received adjuvant osimertinib. Median lung V5 and V20 were 37.7% (range: 8.2–61.5%) and 22.0% (range: 3.4–36.2%), respectively.

**Table 1:** Patient and treatment characteristics

Characteristics		
Age (years), median (range)	67 (25–84)	
Sex	Male Female	99 31
ECOG PS at the initiation of radiotherapy	0/1	86/44
Smoking history	Yes/No	109/21
TNM classification (UICC 8th)		
Stage T stage N stage	IIB/IIIA/IIIB/IIIC 1/2/3/4/X 0/1/2/3	3/51/59/17 26/34/23/44/3 6/14/71/39
Pathology	Adenocarcinoma Squamous cell carcinoma Others	73 38 19
Clinically actionable genetic mutations	Yes/No/Unknown	26/84/20
PD-L1 TPS	< 1% 1%–49%	54 76
ILA score	0 1 2	111 16 3
Chemotherapy	Cisplatin + Vinorelbine Low dose weekly carboplatin Others None	87 22 16 5
Radiation dose	66 Gy in 33 fractions 60 Gy in 30 fractions Others	18 106 6
Radiation technique	3DCRT/IMRT	63/67
Use of intentional internal high-dose VMAT	Yes/No	20/110
Administration of durvalumab	Yes/No	75/55
Lung V5 (%), median (range)	37.7 (8.2–61.5)	
Lung V20 (%), median (range)	22.0 (3.4–36.2)	
Heart volume (cc), median (range)	644 (433–1184)	
Mean heart dose (Gy), median (range)	7.5 (0.1–43.6)	
Max heart dose (Gy), median (range)	63.1 (0.4–75.1)	

Abbreviations: ECOG, Eastern Cooperative Oncology Group; ILA, interstitial lung abnormality; IMRT, intensity-modulated radiotherapy; PD-L1, programmed cell death ligand 1; PS, performance status; TPS, tumour proportion score; VMAT, volumetric modulated arc therapy; 3DCRT, three-dimensional conformal radiation therapy

The median OS was 36 months (range: 2–66 months) at a median follow-up period of 37 months (2–72 months). At the time of the data cut-off, 54 (41.5%) patients died, among whom 49 died of lung cancer progression. Durvalumab was administered to 75 (57.7%) patients. The remaining five patients died, among whom two died of radiation pneumonitis, one each of drug-induced pneumonia, ischaemic heart disease, and nontuberculous mycobacterium tuberculosis. Seventy-nine (60.8%) patients relapsed; the first recurrence site was in-field in 18 patients, distant metastases in 20, and both in 41. Among the 59 (45.4%) patients who experienced local recurrence during the observation period, 23 had a recurrence of the only primary tumour, 20 of only lymph node metastasis, and 16 of both primary and lymph node metastases.

**Table 2:** Initial salvage treatment for 78 patients who experienced recurrence after definitive therapy

Treatment characteristic	No. (%)
Chemotherapy	16 (20.5%)
Immunotherapy	15 (19.2%)
Tyrosine kinase inhibitors	14 (17.9%)
Chemotherapy + Immunotherapy	10 (12.8%)
Radiotherapy	6 (7.7%)
Surgery	4 (5.1%)
Concurrent chemoradiotherapy	2 (2.6%)
Radiotherapy followed by Tyrosine kinase inhibitors	1 (1.3%)
Radiotherapy followed by Chemotherapy	1 (1.3%)
Radiotherapy followed by Chemotherapy + Immunotherapy	1 (1.3%)
No treatment	8 (10.3%)

**Fig. 3:**
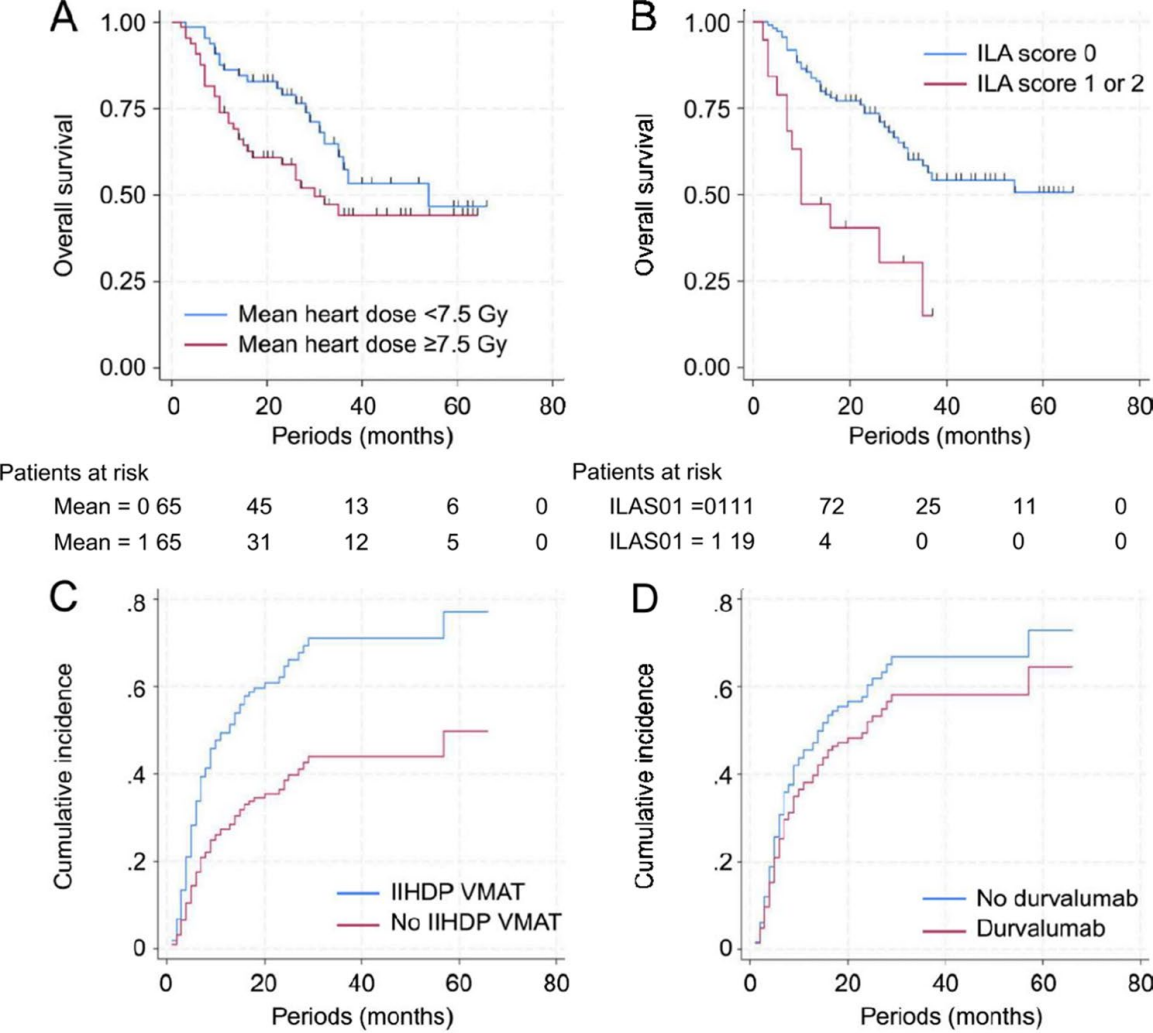
Kaplan–Meier curves of the OS of patients with LA-NSCLC. Kaplan–Meier curves of the OS based on (**a**) mean heart dose and (**b**) ILA scores. Differences in cumulative recurrence rates (**c**) with and without IIHD VMAT and (**d**) with and without durvalumab. OS, overall survival; IIHD VMAT, internal high-dose volumetric modulated arc therapy; ILA, interstitial lung abnormality

The initial salvage treatments are presented in Table 2. Among the 78 (60.0%) patients who relapsed, the first salvage treatment was chemotherapy in 16, immunotherapy in 15, tyrosine kinase inhibitors in 14, chemotherapy + immunotherapy in 10, CCRT in 2, radiotherapy in 6, radiotherapy followed by tyrosine kinase inhibitors in 1, radiotherapy followed by chemotherapy in 1, radiotherapy followed by chemotherapy + immunotherapy in 1, surgery only in 4, and no treatment in 8 patients. Twenty-seven (20.8%) patients had radiation pneumonitis grade ≥ 2; additionally, 19, 6, and 2 patients had grade 2, 3, and 5 radiation pneumonitis, respectively. The multivariate analysis of risk factors for radiation pneumonitis grade ≥ 2 revealed that only an ILA score ≥ 1 was a significant independent predictor. These results are summarized in Supplementary Table 1. The ILA scores of the two patients with grade 5 radiation pneumonitis was 1 and 2, respectively. Furthermore, among the patients with radiation pneumonitis grade ≥ 2, 16 relapsed, and in 3 (18.8%) of these patients, no salvage treatment was available due to radiation pneumonitis. Only two (10.0%) patients who received IIHD VMAT had radiation pneumonitis grade ≥ 2, and both had grade (2) Notably, no other side effects attributable to IIHD VMAT were observed. Tables 3 and 4 represents the results of the univariate and multivariate analyses for the survival outcomes in all patients, respectively. On multivariate analysis, an ILA score ≥ 1 was identified as a significant prognostic factor for OS, while a mean heart dose ≥ 7.5 Gy showed a trend toward significance. Table 5 shows a comparison of patient and treatment characteristics in patients with ILA 0 and 1–2. Figure 3 shows the Kaplan–Meier survival curves for OS divided by the ILA score and mean heart dose. The 2-year survival rates were 73.6% (95% CI: 65.6–82.6%) in the patients with ILA score of 0 (vs. 40.6% [95% CI: 23.1–71.2%] in the others), and 58.8% [95% CI: 47.7–72.4%] in the patients whose mean heart dose ≥ 7.5 Gy (vs. 78.9% [95% CI: 69.2–89.9%] in the others). Furthermore, an additional competing risk analysis revealed that the ILA score was a strong predictor of grade ≥ 2 radiation pneumonitis. Patients with an ILA score ≥ 1 had a significantly higher risk compared to those with a score of 0 (HR: 6.704; 95% CI: 3.198–14.05; *p* < 0.001). Furthermore, high ILA scores were significantly associated with higher CI-IFR; IIHD VMAT, and poor PS at baseline were correlated with lower CI-R, and higher CI-DM in the multivariate analyses, respectively. Durvalumab administration was marginally associated with a lower CI-R. The median OS in the patients who received durvalumab and those who did not were 37 months (3–54 months) vs. 35 months (2–66 months), respectively. The estimated 2-year CI-R were 61.0% (95% CI: 46.0–76.0%) in the durvalumab group and 45.9% (95% CI: 32.7–59.2%) in the non-durvalumab group.

**Table 3:** Univariate analyses of OS, CI-CSD, CI-R, CI-DM and CI-IFR

**OS** **HR (95% CI)**, *** p*-value**	**CI-CSD** **HR (95% CI)**, *** p*-value**	**CI**-***R*** **HR (95% CI)**, *** p*-value**	**CI-DM** **HR (95% CI)**, *** p*-value**	**CI-IFR** **HR (95% CI)**, *** p*-value**
Age ≥ 67 years				
1.523 (0.887–2.615), *p* = 0.128	1.309 (0.753–2.275), *p* = 0.340	0.942 (0.611–1.451), *p* = 0.780	0.699 (0.423–1.155), *p* = 0.160	1.518 (0.916–2.515), *p* = 0.110
Sex (vs. female)				
**2.196 (1.036**–**4.659)**,*** p = 0.040***	**1.866 (0.906–3.846)**,*** p = 0.091***	1.137 (0.706–1.831), *p* = 0.600	1.393 (0.768–2.528), *p* = 0.280	1.188 (0.665–2.122), *p* = 0.560
ECOG PS at baseline (vs. 0)				
**1.720 (1.000–2.959)**,*** p = 0.050***	1.349 (0.776–2.345), *p* = 0.290	0.760 (0.480–1.205), *p* = 0.240	**0.511 (0.282**–**0.928)**,*** p = 0.027***	1.046 (0.628–1.742), *p* = 0.860
Smoking history				
**2.886 (1.042**–**7.997)**,*** p = 0.042***	**2.483 (0.921**–**6.691)**,*** p = 0.072***	0.967 (0.594–1.573), *p* = 0.890	1.213 (0.630–2.336), *p* = 0.560	1.231 (0.646–2.347), *p* = 0.530
Stage ≥ IIIB				
1.031 (0.595–1.787), *p* = 0.914	1.022 (0.581–1.799), *p* = 0.940	1.194 (0.774–1.842), *p* = 0.420	1.144 (0.696–1.881), *p* = 0.600	1.099 (0.666–1.813), *p* = 0.710
T stage ≥ 3				
**1.765 (1.015**–**3.069)**,*** p = 0.044***	**1.756 (0.990–3.115)**,*** p = 0.054***	0.917 (0.595–1.413), *p* = 0.690	0.866 (0.527–1.424), *p* = 0.570	1.043 (0.634–1.714), *p* = 0.870
N3				
0.880 (0.489–1.580), *p* = 0.668	0.813 (0.445–1.485), *p* = 0.500	1.114 (0.692–1.792), *p* = 0.660	1.351 (0.799–2.285), *p* = 0.260	0.881 (0.507–1.528), *p* = 0.650
Clinically actionable genetic mutation				
**0.260 (0.093**–**0.730)**,*** p = 0.010***	**0.315 (0.115**–**0.865)**,*** p = 0.025***	1.408 (0.879–2.255), *p* = 0.150	1.448 (0.854–2.455), *p* = 0.170	1.011 (0.545–1.876), *p* = 0.970
Negative PD-L1 expression				
1.005 (0.584–1.731), *p* = 0.985	1.139 (0.656–1.978), *p* = 0.640	1.059 (0.675–1.661), *p* = 0.800	0.936 (0.560–1.567), *p* = 0.800	1.012 (0.609–1.679), *p* = 0.960
ILA score ≥ 1 (vs. 0)				
**3.238 (1.718**–**6.102)**,*** p < 0.001***	**2.511 (1.275**–**4.948)**,*** p = 0.008***	1.290 (0.736–2.262), *p* = 0.370	1.562 (0.814–2.997), *p* = 0.180	**2.097 (1.176**–**3.740)**,*** p = 0.012***
IMRT^¦^ (vs. 3DCRT)				
1.083 (0.628–1.866), *p* = 0.774	0.883 (0.504–1.550), *p* = 0.670	**0.594 (0.382**–**0.924)**, *p* = 0.021	0.827 (0.503–1.358), *p* = 0.450	0.941 (0.573–1.544), *p* = 0.810
Intentional internal high-dose VMAT				
0.897 (0.381–2.113), *p* = 0.804	0.797 (0.320–1.986), *p* = 0.630	**0.445 (0.210**–**0.943)**, *p* = 0.035	0.477 (0.194–1.175), *p* = 0.110	0.575 (0.257–1.284), *p* = 0.180
Durvalumab administration				
0.811 (0.473–1.393), *p* = 0.448	0.694 (0.396–1.216), *p* = 0.200	0.700 (0.455–1.077), *p* = 0.110	1.015 (0.613–1.680), *p* = 0.960	0.752 (0.456–1.240), *p* = 0.260
Lung V5 ≥ 37.7%				
1.092 (0.640–1.863), *p* = 0.746	1.015 (0.585–1.763), *p* = 0.960	0.941 (0.610–1.450), *p* = 0.780	1.375 (0.839–2.252), *p* = 0.210	1.000 (0.671–1.802), *p* = 0.710
Lung V20 ≥ 22.0%				
0.963 (0.564–1.645), *p* = 0.890	1.005 (0.579–1.744), *p* = 0.990	1.407 (0.909–2.177), *p* = 0.130	1.324 (0.807–2.172), *p* = 0.270	1.212 (0.738–1.991), *p* = 0.450
Heart volume ≥ 644 cc				
1.221 (0.713–2.091), *p* = 0.466	1.042 (0.601–1.807), *p* = 0.880	0.885 (0.575–1.364), *p* = 0.580	1.012 (0.617–1.660), *p* = 0.960	0.876 (0.533–1.438), *p* = 0.600
Mean heart dose ≥ 7.5 Gy				
**1.686 (0.979–2.903)**,*** p = 0.060***	**1.626 (0.931–2.838)**,*** p = 0.087***	1.311 (0.851–2.020), *p* = 0.220	1.272 (0.776–2.087), *p* = 0.340	1.303 (0.796–2.134), *p* = 0.290
Max heart dose ≥ 63.1 Gy				
1.187 (0.695–2.027), *p* = 0.530	1.111 (0.640–1.929), *p* = 0.710	1.179 (0.766–1.813), *p* = 0.450	1.080 (0.660–1.769), *p* = 0.760	1.202 (0.733–1.969), *p* = 0.470

Abbreviations: CI-CSD, cumulative incidence of cancer-specific death; CI-DM, cumulative incidence of distant metastasis; CI-IFR, cumulative incidence of in-field recurrence; CI-R, cumulative incidence of recurrence; ECOG, Eastern Cooperative Oncology Group; ILA, interstitial lung abnormality; IMRT, intensity-modulated radiotherapy; OS, overall survival; PD-L1, programmed cell death ligand 1; PS, performance status; VMAT, volumetric modulated arc therapy; 3DCRT, three-dimensional conformal radiation therapy

**Table 4:** Multivariate analyses of OS, CI-CSD, CI-R, CI-DM and CI-IFR

OS HR (95% CI), *p*-value	CI-CSD HR (95% CI), *p*-value	CI-*R* HR (95% CI), *p*-value	CI-DM HR (95% CI), *p*-value	CI-IFR HR (95% CI), *p*-value
Sex (vs. female)				
1.901 (0.733–4.934), *p* = 0.187	1.449(0.652–3.221) *p* = 0.360			
ECOG PS at baseline (vs. 0)				
1.102 (0.521–2.335), *p* = 0.799			**0.542 (0.297**–**0.989)**,*** p = 0.046***	
Smoking history				
2.094 (0.541–8.105), *p* = 0.285	1.966 (0.558–6.924), *p* = 0.290			
T stage ≥ 3				
1.116 (0.582–2.142), *p* = 0.759	1.207 (0.589–2.473), *p* = 0.610			
Clinically actionable genetic mutation				
0.623 (0.180–2.161), *p* = 0.456	0.644 (0.170–2.448), *p* = 0.520			
ILA score ≥ 1 (vs. 0)				
**2.702 (1.155–6.319)**,*** p = 0.022***	2.004 (0.838–4.793), *p* = 0.120			**2.169 (1.198**–**3.925)**,*** p = 0.011***
Intentional internal high-dose VMAT				
		**0.439 (0.206**–**0.939)**,* p* = 0.034	0.535 (0.215–1.329), *p* = 0.180	0.546 (0.254–1.174), *p* = 0.120
Durvalumab administration				
		0.692 (0.450–1.064), *p* = 0.094		
Mean heart dose ≥ 7.5 Gy				
1.941 (0.986–3.823), *p* = 0.055	1.710 (0.848–3.451), *p* = 0.130			

Abbreviations: CI-CSD, cumulative incidence of cancer-specific death; CI-DM, cumulative incidence of distant metastasis; CI-IFR, cumulative incidence of in-field recurrence; CI-R, cumulative incidence of recurrence; ECOG, Eastern Cooperative Oncology Group; ILA, interstitial lung abnormality; PS, performance status; VMAT, volumetric modulated arc therapy

**Table 5:** Comparison of patient and treatment characteristics between patients with an ILA score of 0 and those with an ILA score of 1 or 2

Characteristics		ILA 0 (*N* = 111)	ILA 1 or 2 (*N* = 19)	*p*-value
Age (years), median (range)		65 (25–84)	71 (58–82)	*p* = 0.004
Sex	Male	81	18	*p* = 0.040
	Female	30	1	
ECOG PS at the initiation of radiotherapy	0/1	81/30	5/14	*p* < 0.001
Smoking history	Yes/No	90/21	19/0	*p* = 0.038
TNM classification (UICC 8th)				
Stage	IIB/IIIA/IIIB/IIIC	3/45/48/15	0/6/11/2	*p* = 0.607
T stage	1/2/3/4/X	22/30/18/38/3	4/4/5/6	*p* = 0.788
N stage	0/1/2/3	6/12/60/33	0/2/11/6	*p* = 0.779
Pathology	Adenocarcinoma	62	11	*p* = 0.104
	Squamous cell	30	8	
	carcinoma	19	0	
	Others			
Clinically actionable genetic mutations	Yes/No/Unknown	25/70/16	1/14/4	*p* = 0.206
PD-L1 TPS	< 1%	51	3	*p* = 0.014
	1%–49%	60	16	
Chemotherapy	Cisplatin + Vinorelbine	77	10	*p* = 0.276
	Low dose weekly carb oplatin	16	6	
	Others	16	36	
	None	2	0	
Radiation dose	66 Gy in 33 fractions	17	1	*p* = 0.261
	60 Gy in 30 fractions	88	18	
	Others	6	0	
Radiation technique	3DCRT/IMRT	58/53	5/14	*p* = 0.037
Use of intentional internal high-dose VMAT	Yes/No	16/95	4/15	*p* = 0.459
Administration of durvalumab	Yes/No	65/46	10/9	*p* = 0.629
Lung V5 (%), median (range)		36.4 (8.2–61.5)	45.1 (23.0-60.9)	*p* = 0.005
Lung V20 (%), median (range)		21.7 (3.4–33.2)	25.7 (12.9–36.2)	*p* = 0.043
Heart volume (cc), median (range)		643 (433–1184)	668 (480–908)	*p* = 0.377
Mean heart dose (Gy), median (range)		5.6 (0.1–43.6)	11.4 (0.7–31.6)	*p* = 0.014
Max heart dose (Gy), median (range)		62.8 (0.4–75.1)	65.0 (4.3–66.9)	*p* = 0.102

Abbreviations: ECOG, Eastern Cooperative Oncology Group; ILA, interstitial lung abnormality; IMRT, intensity-modulated radiotherapy; PD-L1, programmed cell death ligand 1; PS, performance status; TPS, tumour proportion score; VMAT, volumetric modulated arc therapy; 3DCRT, three-dimensional conformal radiation therapy

Univariate and multivariate results of subgroup analyses of the patients with negative PD-L1 expression are presented in Supplementary Tables 2 and 3, respectively.

In the patients with negative PD-L1 expression, only three patients had an ILA score ≥ 1, and nine underwent IIHD VMAT; therefore, they were excluded from the analysis. Notably, all nine patients who received IIHD VMAT in the patients with negative PD-L1 expression survived without relapse during the median follow-up time of 18 months. In the multivariate analyses, clinically actionable genetic mutations were marginally associated with CI-DM [HR: 2.001 (95% CI: 0.929–4.314), *p* = 0.077]. IMRT was associated with a significantly lower CI-R [HR: 0.442 (95% CI: 0.217–0.900), *p* = 0.024], and high mean heart dose was marginally associated with a higher CI-R and CI-IFR [HR: 1.764 (95% CI: 0.901–3.454), *p* = 0.098 and HR: 2.066 (95% CI: 0.931–4.584), *p* = 0.074].

## Discussion

This study’s findings demonstrated that a high ILA score was a significant unfavourable factor of OS, while a high mean heart dose showed a trend toward a negative impact in patients with LA-NSCLC with negative or low PD-L1 expression. In a previous study [13], ILA score was significantly associated with OS in patients with LA-NSCLC receiving CCRT plus adjuvant durvalumab. Our study indicated that the ILA score was a significant factor of OS not only in patients who received adjuvant durvalumab but also in those with negative or low PD-L1 expression with/without adjuvant durvalumab. The ILA score is a predictive factor of radiation pneumonitis requiring steroid administration [18]. In this study, ILA scores were more strongly correlated with OS than with cause-specific survival, which suggests that patients with high ILA scores die of causes other than lung cancer. Two patients who had grade 5 radiation pneumonitis with ILA scores of 1 and 2, respectively, and three who experienced radiation pneumonitis grade ≥ 2 could not receive post-treatment due to radiation pneumonitis, which implies strong lung toxicities in those with high ILA scores, resulting in the deterioration of PS. Furthermore, the use of glucocorticoids for the management of radiation pneumonitis may affect the tumour immune microenvironment and has been associated in some studies with reduced efficacy of immune checkpoint inhibitors (ICIs) [19–21]. However, recent evidence suggests that this effect may depend on the dose intensity and timing of glucocorticoid use; for example, one study reported that high peak doses were associated with worse overall survival, while cumulative dose had no significant impact [22]. Therefore, the relationship between glucocorticoid use and ICI efficacy remains controversial. Regarding heart radiation dose, in the RTOG0617 trial, the dose was significantly correlated with OS [12]. Nonetheless, this trial was conducted before the introduction of adjuvant durvalumab. Our study demonstrated that the heart dose showed a trend toward an association with OS in real-world data after the PACIFIC trial. In this study, only one patient died of ischaemic heart disease; however, the true morbidity of heart disease associated with radiotherapy tended to be underestimated [23]. Furthermore, higher heart doses were reported to increase cardiac toxicities [24–26] and immunosuppression by killing lymphocytes [27–29]. Therefore, lymphopenia, caused by increased radiation doses delivered to the heart, would be a critical risk factor in the immunoradiotherapy era. At our institution, we are currently planning a prospective clinical trial to evaluate the feasibility and effectiveness of heart-sparing radiotherapy techniques in patients with locally advanced NSCLC receiving chemoradiotherapy. This trial will aim to reduce cardiac radiation exposure while maintaining optimal tumour coverage. In addition to clinical outcomes, comprehensive cardiac monitoring will be implemented, including echocardiographic evaluation of global longitudinal strain and serial assessment of cardiac biomarkers such as troponin and brain natriuretic peptide (BNP). These assessments will allow for early detection of cardiac dysfunction and provide insight into the relationship between heart dose and cardiotoxicity in the context of multimodal treatment.

Patients with stage III NSCLC with negative or low PD-L1 expression who received CCRT and adjuvant durvalumab were reported to have a worse prognosis than did those with high expression of PD-L1 [1, 5, 10, 13]. However, to our knowledge, no real-world data provide prognostic analyses of definitive chemoradiotherapy with or without adjuvant durvalumab only in patients with LA-NSCLC with negative or low PD-L1 expression to date. Although studies have examined the efficacy of durvalumab administration in patients with negative or low PD-L1 expression [4, 5, 11], other prognostic factors remain unknown. In our study, durvalumab administration was a marginally favourable prognostic factor for CI-Rin patients with PD-L1 TPS ≤ 49%, and did not correlate with OS and CI-CSD, which is consistent with the results of the subgroup analysis of the 5-year results of the PACIFIC trial [4]. That might be because post-treatment, including tyrosine kinase inhibitor treatment, was highly effective. The LAURA trial showed that osimertinib after definitive chemoradiotherapy for patients with EGFR mutated LA-NSCLC significantly prolonged PFS, and adjuvant osimertinib rather than durvalumab would be preferred in such patients [30].

The use of IIHD VMAT was significantly associated with favourable CI-R. In the RTOG0617 study [31, 32], dose escalation was ineffective for CCRT in patients with LA-NSCLC. Nevertheless, dose escalation using high-precision radiotherapy techniques has recently been attempted. In the PET-Boost trial, a phase II randomised clinical trial [33], 18% of the patients experienced grade 5 adverse events. In the study by Hallqvist et al. [34], dose escalation to 84 Gy resulted in a high rate of treatment-related deaths due to oesophageal perforation and pneumonitis. Additionally, in the KROG0903 trial [35], accelerated hypofractionated radiotherapy with 60 Gy in 25 fractions did not improve local tumour control rates or PFS compared with conventional fractionated irradiation. In contrast, IIHD VMAT increases the dose only at the centre of the tumour without increasing the tumour’s peripheral dose using the SIB technique. This enables the bioequivalent dose inside the tumour to be considerably higher than a simple dose escalation, while the dose to organs at risk can be spared. The incidence of radiation pneumonitis grade ≥ 2 was low in patients who underwent IIHD VMAT, and no other serious adverse events, including oesophageal perforation, were observed. However, the number of patients receiving IIHD VMAT in this study was small; therefore, additional research with a larger sample size is required.

Our study has several potential limitations. First, this study used maximum and mean dose for DVH parameters of the heart; however, the optimal parameter is unclear [36–38]. In addition, cardiac laboratory markers (e.g., troponin, BNP) were not routinely collected in this retrospective study. Consequently, future studies incorporating both regional cardiac dose analysis and cardiac biomarkers are warranted to better understand radiation-induced cardiac injury and its prognostic impact. Second, molecular testing was not uniformly performed prior to treatment in all patients, which limits the generalizability and interpretability of analyses related to genetic mutations. Third, this was a retrospective study; therefore, unknown biases could be included.

## Conclusion

ILA scores significantly impacted OS, while a mean heart dose showed a trend toward a negative impact on OS in patients with negative or low PD-L1 expression and LA-NSCLC according to real-world data obtained after the PACIFIC trial. Moreover, the heart dose was marginally associated with OS in Asian patients after the PACIFIC trial. However, a more comprehensive analysis of cardiac anatomy is needed in the future.

## Supplementary Information

Below is the link to the electronic supplementary material.

Supplementary Material 1

Supplementary Material 2

Supplementary Material 3

## Data Availability

The datasets generated and analysed during the current study are not publicly available for reasons of privacy protection but are available from the corresponding author on reasonable request.

## Abbreviations

BNP: Brain natriuretic peptide
CCRT: Concurrent chemoradiotherapy
CI-CSD: Cumulative incidence of cancer-specific death
CI-DM: Cumulative incidence of distant metastasis
CI-IFR: Cumulative incidence of in-field recurrence
CI-R: Cumulative incidence of recurrence
CT: Computed tomography
CTV: Clinical target volume
DVH: Dose volume histograms
ICI: Immune checkpoint inhibitor
IIHD VMAT: Intentional internal high-dose volumetric modulated arc therapy
ILA: Interstitial lung abnormality
IMRT: Intensity-modulated radiation therapy
LA-NSCLC: Locally advanced non-small cell lung cancer
OS: Overall survival
PD-L1: Programmed cell death ligand 1
PFS: Progression-free survival
PS: Performance status
TPS: Tumour proportion score
