# Supplementary material for: Locally advanced non-small cell lung cancer with negative or low programmed death ligand 1 expression: a prognostic factor analysis of real-world data after the PACIFIC trial
Source: Radiat Oncol. 2025 Oct 16;20:155. doi: 10.1186/s13014-025-02733-5 (PMC12529796; doi:10.1186/s13014-025-02733-5)
Supplement: Supplementary file 1 — Supplementary Material 1 [file 13014_2025_2733_MOESM1_ESM.docx]

**Supplementary Table 1.** Univariate and multivariate analyses of predictors for radiation pneumonitis ≥grade 2.

|  |  |  |
| --- | --- | --- |
|  | **Univariate analyses**  HR (95% CI), p-value | **Multivariate analyses**  HR (95% CI), p-value |
| Age ≥67 years | 1.923 (0.857–4.316), p = 0.113 |  |
| Sex (vs. female) | 1.020 (0.415–2.501), p = 0.965 |  |
| ECOG PS at baseline (vs. 0) | 1.662 (0.766–3.605), p = 0.199 |  |
| Smoking history | 1.491 (0.459–4.840), p = 0.506 |  |
| Stage ≥IIIB | 1.646 (0.721–3.759), p = 0.236 |  |
| T stage ≥3 | 0.747 (0.344–1.622), p = 0.460 |  |
| N3 | 1.149 (0.497–2.653), p = 0.745 |  |
| Genetic mutation | 1.354 (0.584–3.143), p = 0.480 |  |
| Negative PD-L1 expression | 0.624 (0.273–1.422), p = 0.262 |  |
| ILA score ≥1 (vs. 0) | **6.379 (2.945**–**13.818), p < 0.001** | **4.985 (2.236**–**11.175), p < 0.001** |
| IMRT^¦^ (vs. 3DCRT) | 1.547 (0.698–3.430), p = 0.282 |  |
| IIHD VMAT | 0.762 (0.235–2.467), p = 0.650 |  |
| Durvalumab administration | 0.808 (0.375–1.738), p = 0.585 |  |
| Lung V5 ≥37.7% | **2.881 (1.224–6.784), p = 0.015** | 2.021 (0.883–4.626), p = 0.096 |
| Lung V20 ≥22.0% | **2.834 (1.210–6.638), p = 0.016*** |  |
| Heart volume ≥644 cc | 1.308 (0.602–2.841), p = 0.498 |  |
| Mean heart dose ≥7.5 Gy | 1.943 (0.870–4.337), p = 0.105 |  |
| Max heart dose ≥63.1 Gy | **2.816 (1.178–6.728), p = 0.020** | 1.934 (0.779–4.798), p = 0.155 |

* In the univariate analyses, both lung V5 and V20 were significant predictors of radiation pneumonitis ≥grade 2. However, due to clear collinearity between these two factors, only lung V5, which had a higher hazard ratio, was included in the multivariate analysis.

Abbreviations: ECOG, Eastern Cooperative Oncology Group; ILA, interstitial lung abnormality; IMRT, intensity-modulated radiotherapy; PD-L1, programmed cell death ligand 1; PS, performance status; IIHD VMAT, intentional internal high-dose volumetric modulated arc therapy; 3DCRT, three-dimensional conformal radiation therapy
