# Supplementary material for: Locally advanced non-small cell lung cancer with negative or low programmed death ligand 1 expression: a prognostic factor analysis of real-world data after the PACIFIC trial
Source: Radiat Oncol. 2025 Oct 16;20:155. doi: 10.1186/s13014-025-02733-5 (PMC12529796; doi:10.1186/s13014-025-02733-5)
Supplement: Supplementary file 2 — Supplementary Material 2 [file 13014_2025_2733_MOESM2_ESM.docx]

**Supplementary Table 2.** Univariate results of subgroup analyses of the patient group with negative PD-L1 expression

**OS** **CI-CSD**  **CI-R**  **CI-DM**  **CI-IFR**

HR (95%CI), *p* value HR (95%CI), *p* value HR (95%CI), *p* value HR (95%CI), *p* value HR (95%CI), *p* value


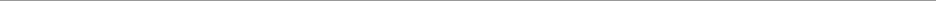


**Univariate analyses**

Age ≥64

1.323 (0.569-3.073), *p* = 0.516 1.164 (0.502-2.698), *p* = 0.720 1.122 (0.571-2.202), *p* = 0.740 0.849 (0.390-1.846), *p* = 0.680 1.394 (0.624-3.115), *p* = 0.420

Sex (vs Female)

1.792 (0.598-5.373), *p* = 0.298 1.654 (0.571-4.789), *p* = 0.350 0.842 (0.416-1.704), *p* = 0.630 0.885 (0.377-2.078), *p* = 0.780 0.771 (0.308-1.928), *p* = 0.580

ECOG PS at baseline (vs 0)

1.052 (0.408-2.708), *p* = 0.917 0.817 (0.298-2.240), *p* = 0.690 0.677 (0.290-1.583), *p* = 0.370 0.431 (0.143-1.295), *p* = 0.130 0.684 (0.258-1.814), *p* = 0.450

Smoking history

3.550 (0.476-26.465), *p* = 0.216 3.327 (0.513-21.580), *p* = 0.210 1.584 (0.631-3.974), *p* = 0.330 1.766 (0.515-6.058), *p* = 0.370 3.506 (0.508-24.20), *p* = 0.200

Stage ≥ IIIB

1.490 (0.606-3.662), *p* = 0.385 1.349 (0.560-3.250), *p* = 0.510 1.227 (0.630-2.391), *p* = 0.550 0.810 (0.378-1.736), *p* = 0.590 0.909 (0.417-1.980), *p* = 0.810

T stage ≥ 3

**2.592 (1.009-6.659), *p* = 0.048** **2.352 (0.924-5.985), *p* = 0.073** 1.117 (0.571-2.184), *p* = 0.750 0.840 (0.388-1.820), *p* = 0.660 1.507 (0.670-3.393), *p* = 0.320

N3

0.820 (0.332-2.023), *p* = 0.666 0.658 (0.262-1.653), *p* = 0.370 0.763 (0.364-1.600), *p* = 0.470 0.996 (0.443-2.236), *p* = 0.990 0.582 (0.243-1.392), *p* = 0.220

Clinically actionable genetic mutation

0.288 (0.038-2.190), *p* = 0.229 0.312 (0.057-1.682), *p* = 0.180 1.561 (0.821-2.968), *p* = 0.170 **2.300 (1.137-4.653), *p* = 0.021** 0.668 (0.161-2.761), *p* = 0.580

IMRT (vs 3DCRT)

0.672 (0.284-1.589), *p* = 0.365 0.577 (0.240-1.386), *p* = 0.220 **0.432 (0.214-0.872), *p* = 0.019** 0.594 (0.269-1.311), *p* = 0.200 0.785 (0.353-1.744), *p* = 0.550

Durvalumab administration

1.462 (0.559-3.828), *p* = 0.439 1.315 (0.488-3.547), *p* = 0.590 0.836 (0.417-1.674), *p* = 0.610 1.233 (0.528-2.884), *p* = 0.630 1.204 (0.497-2.918), *p* = 0.680

Lung V5 ≥ 36.1%

0.856 (0.368-1.989), *p* = 0.718 0.739 (0.314-1.740), *p* = 0.490 0.907 (0.461-1.781), *p* = 0.780 1.063 (0.491-2.301), *p* = 0.880 1.265 (0.566-2.824), *p* = 0.570

Lung V20 ≥ 20.1%

0.748 (0.321-1.742), *p* = 0.501 0.850 (0.371-1.949), *p* = 0.700 0.991 (0.504-1.948), *p* = 0.980 0.979 (0.451-2.125), *p* = 0.960 0.822 (0.373-1.810), *p* = 0.630

Heart volume ≥ 638 cc

0.816 (0.351-1.895), *p* = 0.636 0.706 (0.305-1.632), *p* = 0.420 0.907 (0.465-1.771), *p* = 0.780 1.039 (0.481-2.246), *p* = 0.920 0.662 (0.301-1.454), *p* = 0.300

Mean heart dose ≥ 5.15 Gy

1.496 (0.640-3.496), *p* = 0.353 1.740 (0.753-4.024), *p* = 0.200 **1.826 (0.923-3.611), *p* = 0.083** 1.442 (0.669-3.107), *p* = 0.350 **1.997 (0.896-4.448), *p* = 0.091**

Max heart dose ≥ 62.6 Gy

0.535 (0.225-1.269), *p* = 0.156 0.607 (0.257-1.432), *p* = 0.250 1.011 (0.516-1.980), *p* = 0.970 0.956 (0.441-2.074), *p* = 0.910 0.688 (0.305-1.551), *p* = 0.370


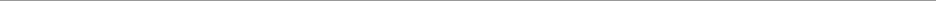


Abbreviations: CI-CSD, cumulative incidence of cancer-specific death; CI-DM, cumulative incidence of distant metastasis; CI-IFR, cumulative incidence of in-field recurrence; CI-R, cumulative incidence of recurrence; ECOG, Eastern Cooperative Oncology Group; IMRT, intensity-modulated radiotherapy; OS, overall survival; PD-L1; programmed cell death ligand 1; PS, performance status; 3DCRT, three-dimensional conformal radiation therapy
