# Supplementary material for: Locally advanced non-small cell lung cancer with negative or low programmed death ligand 1 expression: a prognostic factor analysis of real-world data after the PACIFIC trial
Source: Radiat Oncol. 2025 Oct 16;20:155. doi: 10.1186/s13014-025-02733-5 (PMC12529796; doi:10.1186/s13014-025-02733-5)
Supplement: Supplementary file 3 — Supplementary Material 3 [file 13014_2025_2733_MOESM3_ESM.docx]

**Supplementary Table 3.** Multivariate subgroup analyses of the patient group with negative PD-L1 expression

**OS** **CI-CSD**  **CI-R** **CI-DM**  **CI-IFR**

HR (95%CI), *p* value HR (95%CI), *p* value HR (95%CI), *p* value HR (95%CI), *p* value HR (95%CI), *p* value


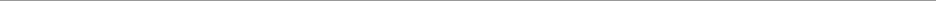


**Multivariate analyses**

ECOG PS at baseline (vs 0)

0.464 (0.156-1.380), *p* = 0.170

Smoking history

3.723 (0.568-24.39), *p* = 0.170

T stage ≥ 3

2.265 (0.799-6.424), *p* = 0.124 2.013 (0.693-5.845), *p* = 0.200

Clinically actionable genetic mutation

0.288 (0.038-2.200), *p* = 0.230 0.314 (0.066-1.503), *p* = 0.150 2.001 (0.929-4.314), *p* = 0.077

IMRT (vs 3DCRT)

**0.442 (0.217-0.900), *p* = 0.024**

Mean heart dose ≥ 5.15 Gy

1.764 (0.901-3.454), *p* = 0.098 2.066 (0.931-4.584), *p* = 0.074


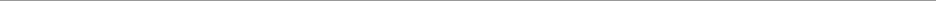


Abbreviations: CI-CSD, cumulative incidence of cancer-specific death; CI-DM, cumulative incidence of distant metastasis; CI-IFR, cumulative incidence of in-field recurrence; CI-R, cumulative incidence of recurrence; ECOG, Eastern Cooperative Oncology Group; IMRT, intensity-modulated radiotherapy; OS, overall survival; PD-L1; programmed cell death ligand 1; PS, performance status; 3DCRT, three-dimensional conformal radiation therapy
